# Electromagnetic Fields Modify Redox Balance in the Rat Gastrointestinal Tract

**DOI:** 10.3389/fpubh.2021.710484

**Published:** 2021-09-13

**Authors:** Karolina Sieroń, Katarzyna Knapik, Grzegorz Onik, Ewa Romuk, Ewa Birkner, Sebastian Kwiatek, Aleksander Sieroń

**Affiliations:** ^1^Chair of Physiotherapy, Department of Physical Medicine, School of Health Sciences in Katowice, Medical University of Silesia in Katowice, Katowice, Poland; ^2^Department of Biochemistry, School of Medicine with the Division of Dentistry in Zabrze, Medical University of Silesia in Katowice, Katowice, Poland; ^3^Division of Internal Diseases Oncology, Gastroenterology, Angiology, Department of Cardiology Intensive Care, Hospital of the Ministry of the Interior and Administration in Katowice, Katowice, Poland; ^4^Department of Physiotherapy, Jan Dlugosz University in Czestochowa, Czestochowa, Poland

**Keywords:** oxidative stress, redox balance, electromagnetic fields, gastrointestinal tract, mobile phones

## Abstract

**Objective:** The aim of the study was to assess the influence of electromagnetic fields with divergent physical properties on the prooxidative and antioxidative balances in homogenates of the tongue, salivary glands, esophagus, stomach, and small and large intestines of rats.

**Material and Methods:** Forty rats were randomly divided into four equal groups, namely, a control group, a group exposed to low-frequency electromagnetic fields (LF-EMFs; frequency: 50 Hz; intensity: 10 kV/m; magnetic induction: 4.3 pT), a group exposed to radiofrequency electromagnetic fields (RF-EMFs) emitted by mobile phones (frequency: 900 MHz), and a group exposed simultaneously to LF-EMFs and RF-EMFs emitted by mobile phones. After 28 consecutive days of the experiment, the following pro- and antioxidative markers were assessed in the gastrointestinal tract homogenates: superoxide dismutase (SOD) and its two isoenzymes (Mn-SOD, Cu,Zn-SOD) catalase (CAT), glutathione peroxidase (GPx), glutathione reductase (GR), glutathione S-transferase (GST), total antioxidative capacity (TAC), total oxidative status (TOS), and malondialdehyde (MDA).

**Results:** In rats exposed to LF-EMFs, higher concentrations of the markers of prooxidant processes, MDA or TOS, were observed in the salivary glands, esophagus, and small intestine homogenates in comparison with the control group. Additionally, in the group of rats opposite to the control, antioxidant activity was observed. The main differences included a higher activity of Cu,Zn-SOD in homogenates of the tongue, salivary glands, and esophagus as well as decreased activity of CAT in homogenates of the tongue, esophagus, and small intestine. In animals exposed to RF-EMFs, the concentration of TOS was higher in the large intestine than in control rats. The main difference of antioxidant activity was presented by decreased Cu,Zn-SOD in homogenates of the salivary glands, stomach, small and large intestine as well as CAT in homogenates of the tongue, esophagus, stomach, and small and large intestine. Moreover, in rats exposed simultaneously to LF-EMFs and RF-EMFs, a lower concentration of TOS was observed. Antioxidant activity was presented by a decreased activity of CAT in homogenates of the tongue, esophagus, stomach, and small and large intestine in comparison to the control group.

**Conclusion:** Among those applied in the study, electromagnetic fields of a low-frequency caused the most significant disturbances of oxidative stress in the rat gastrointestinal tract.

## Introduction

Throughout their lifetime, people are constantly exposed to a wide spectrum of electromagnetic waves with various properties. Electromagnetic fields may be generated by natural or manmade sources. Recently, low-frequency electromagnetic fields (LF-EMFs) and radiofrequency electromagnetic fields (RF-EMFs) have become a commonplace in our environment as a consequence of dynamic technological development. Whereas, LF-EMFs are generated mainly by alternating current power lines and devices powered with alternating current, RF-EMFs are emitted by mobile phones, mobile phone base stations, and wireless LAN.

Despite the ubiquity of electromagnetic field emitters, their influence on living organisms is as of now undetermined. Their potential harmful effects are public health issues, which are being considered in various societies. Mass media have made numerous appeals for a reliable explanation of their influence on human beings and, among other things, how to protect against them. That is why an assessment of how electromagnetic fields affect our world is the subject of numerous research studies.

Epidemiological studies are focused mainly on establishing a connection between electromagnetic fields and the occurrence of cancer. Indeed, pooled analyses of studies proved that prolonged exposure to LF-EMFs increases the risk of leukemia (1–3), but it does not enhance the risk of brain tumors in children (4). Yet, it is suspected that long-lasting exposure to LF-EMFs results in a significantly increased risk of brain tumors (5–7), leukemia (8, 9), kidney (5), liver, and biliary cancers (10) in adults. Nevertheless, some studies do not support such findings (11–13). Furthermore, the oncogenic effect of RF-EMFs is still ambiguous and questionable. In this context, the influence of prolonged usage of mobile phones on brain tumor occurrence still arouses interest. Some research (14–17) has confirmed an increased risk of brain tumor development in people using mobile phones frequently. Furthermore, many studies (18–22) are dedicated to the potential influence of LF-EMFs and RF-EMFs on the endocrine, nervous, cardiovascular, and reproductive systems. Electromagnetic hypersensitivity is also a subject of interest. It may be accompanied by various symptoms, such as paresthesia, asthenia, headaches, and heart palpitations (23).

The potential influence of LF-MF on redox balance has been assessed in numerous research studies (24, 25). Among all tissues of the human body, the brain has the highest sensitivity to oxidative injuries, which is why numerous studies are focused on the influence of LF-EMFs and RF-EMFs on the nervous system. It has been proven that exposure to electromagnetic fields decreases antioxidant activity, leading to oxidative injuries (26–29). In guinea pigs, a 30-day exposure to RF-EMFs resulted in the decreased activity of glutathione (GSH) and catalase (CAT) with a concomitant elevation of malondialdehyde (MDA) in the brain (26). Exposure to LF-EMFs also reduces the activity of CAT and total antioxidant capacity (TAC), with a secondary increase of the MDA, total oxidative status (TOS), and oxidative stress index in rat brains (27). Moreover, it was also proven that antioxidants administered exogenously, e.g., lotus seedpod procyanidins (28) and vitamin E (29), increased the resistance of tissues to electromagnetic-field-mediated injuries. Among gastrointestinal tract tissues, the effects of RF-EMFs action on the liver have been firmly established (30–32). RF-EMFs were proven to enhance prooxidative processes and diminish antioxidant protection in the livers of rats (30, 31) and guinea pigs (32). Also, the application of *N*-acetyl cysteine and epigallocatechin gallate has a protective value (32).

The influence of extrinsic factors on oxidative stress parameters in the gastrointestinal tract still remains unestablished. To our knowledge, none of the previous studies have assessed the influence of LF-EMFs and RF-EMFs on redox balance in the gastrointestinal tract. Because of the complex character of pro- and anti-oxidative reactions, assessing the wide range of oxidative stress markers to obtain reliable results is essential, as we did in our study. What's more, we have also decided to assess two widespread electromagnetic fields of low- and radio-frequency. As organisms may be exposed to the simultaneous action of those electromagnetic fields repeatedly, we have considered such conditions in our study.

Therefore, the aim of the study was to assess the influence of divergent electromagnetic fields on oxidative status parameters in homogenates of the tongue, salivary glands, esophagus, stomach, and the small and large intestines of rats. We assumed that a sustained 4-week exposure to the electromagnetic fields is adequate.

## Materials and Methods

### Animals

The study protocol was approved by the Bioethical Committee for Animal Experimentation of the Medical University of Silesia in Katowice, Poland. All animals received humane care in compliance with the 8th edition of the Guide for the Care and Use of Laboratory Animals published by the National Institute of Health (33).

This experiment included 40 male Wistar rats at the age of 10 weeks and a mean initial body mass of 260–280 g (BMI: 0.50–0.65 g/cm^2^). The animals were obtained and bred at the Institute of Experimental Medicine, Medical University of Silesia in Katowice. During the experiment, the animals were kept in an optimal environment. Mean temperature was 21°C, mean air humidity was 60%, and a 24-h circadian rhythm was maintained. The rats were kept in plastic cages which did not restrict spontaneous activity. Furthermore, the cage construction did not impair the action of the electromagnetic fields. The animals were housed 10 per cage. Each rat ate about 15–30 g of standard laboratory pellet food daily (*Labofed B*, consisting of 8% fat, 25% protein, and 67% carbohydrate) and was provided water *ad libitum*.

### Experiment Design

The experiment lasted 28 consecutive days. Forty rats were randomly divided into four equal groups. In the control rats, sham exposures were performed daily without magnetic fields. They (Group I) remained in plastic cages positioned between two electrodes placed 50 cm apart for 22 h/day with a 2-h break between 8 and 10 a.m. During the sham exposure, magnetic fields were not generated. Rats included in Group II were exposed to LF-EMFs with features similar to those found near current power lines (frequency: 50 Hz; intensity: 10 kV/m; magnetic induction: 4.3 pT). The animals were exposed to electromagnetic fields for 22 h each day. The magnetic fields were turned off between 8 and 10 a.m. Group III consisted of rats exposed to RF-EMFs emitted by a Nokia 5110 mobile phone with a frequency of 900 MHz. The experiment was carried out every day between 9 a.m. and 1 p.m., and also between 2 and 6 p.m. Every 30 min a single connection lasting 15 s was performed, and therefore, 16 connections per day were carried out. The mean power density of the electromagnetic fields was registered, while initializing the connection was 85.3 μW/m^2^ and during the connection, it was 17.0 μW/m^2^. Group IV was exposed simultaneously to LF-EMFs and RF-EMFs. The physical features of the electromagnetic fields were identical to those applied in Group II and Group III.

During the exposure, the animals were housed in a plastic cage positioned between two electrodes placed 50 cm apart. The plastic cage did not impair the electromagnetic field and allowed the free movement of the rats. One electrode received a potential of 5 kV from a high-voltage transformer. The current was generated by a high-voltage generator. The mobile phone was placed directly under the cage. The cage housing the rats was placed on a ground electrode.

The mean power density of the electromagnetic fields was calculated on the basis of 16 consecutive measurements obtained from five sensors located in the cages of the rats. An electromagnetic interference meter TES-92 (serial number 091006768) was used to perform the measurements. The gauge had a calibration certificate (number: LWiMP/W/025/09, given: 04/02/2010) provided by Laboratorium Wzorców i Meterologii Pola Elektromagnetycznego we Wrocławiu (accreditation number: AP078).

After 28 consecutive days of exposure to electromagnetic fields or sham exposures, the rats fasted for 24 h, and then, they were euthanized with a mixture of xylazine (10 mg/kg *i.p*.) and ketamine (100 mg/kg *i.p*.). Next, after having surgically opened the chest through costotomy and collected total amounts of blood (~4 ml) from the left heart ventricle with the use of a 0.5 × 30 mm injection needle and syringe, the abdominal cavity was opened and the following samples of the gastrointestinal tract were taken: the tongue, salivary glands, esophagus, stomach, and the small and large intestines.

After that, tissues were homogenized on ice in short cycles for a few seconds after cutting the organs into small pieces. For homogenization, a 0.9% NaCl solution (homogenizer Potter-Elvehjem PTFE, pestle and glass tube, Sigma-Aldrich) was used. After mechanical homogenization, we used a UP50H ultrasonic processor (Hielscher). In the next step, the homogenates were centrifuged at 3,000 rpm for 10 min. Homogenates were immediately frozen in liquid nitrogen and stored at −80°C until further analysis, for no longer than 30 days.

### Biochemical Measurements

#### Determination of Antioxidative Activity

The superoxide dismutase (SOD) activity and its two isoenzymes, namely manganese-dependent SOD (Mn-SOD) and copper- and zinc-dependent SOD (Cu,Zn-SOD), were assessed using Oyanahui's method (34). Around 0.1 ml of hydroxylamine solution was added to 50 μl of the sample. Fulfillment of 0.2 ml of xanthine oxidase enzymatic solution was proceeded by a 5-min incubation at a temperature of 37°C. Next, the second incubation was performed (duration: 5 min, temperature: 37°C) with the procurement of colored reagent including acetic acid, naftylenodiamin, and sulfanilic acid. Absorbance was investigated with a VICTOR X3, PerkinElmer. Enzymatic activity was expressed in nitrite units (NU) per milligram of protein. In this method, 1 NU means a 50% inhibition of nitrite ion production by SOD. SOD isoenzymes (Mn-SOD and Cu,Zn-SOD) were measured using potassium cyanide as the inhibitor of the Cu,Zn-SOD isoenzyme. The inter- and intra-assay coefficients of variations (CVs) were 2.8 and 5.4%, respectively.

The CAT activity was measured with Aebi's kinetic method (35). The reaction leads to hydrogen peroxide reduction into water and oxygen. The absorbance kinematics was observed with the Shimadzu UV-1700 spectrophotometer at wavelength 240 nm. Enzymatic activity was expressed in units per one gram of protein (IU/g). The inter- and intra-assay CVs were 2.6 and 6.1%, respectively.

Glutathione peroxidase (GPx) activity was determined with Paglia and Valentine's kinetic method (36), which is based on the reaction of GSH with t-butyl oxide. 10 μl of lysate was combined with a reacting mixture including NADPH, GSH, and glutathione reductase (GR). After initial reaction stabilization, the 25 μl t-butyl was added, and absorbance was noted at wavelength 350 nm. The kinematics of absorbance was investigated for 3 min with consecutive analysis every 60 s using a VICTOR X3 device, PerkinElmer. Enzymatic activity was expressed as micromoles of NADPH utilized per minute and normalized to 1 g of protein (IU/g protein). The inter- and intra-assay CVs were 3.3 and 7.4%, respectively.

Glutathione reductase activity was measured with Richterich's kinetic method (37) to assess reduced NAPDH concentration changes. GPx leads to NAPDH reduction. Here 125 μl of 6 mmol/l NADPH solution was added to 25 μl of supernatant. After the initial reaction stabilization, 25 μl of 75 mmol/l glutathione peroxide was added. Absorbance was read with usage of a 355 nm filter. The kinematics of absorbance changes was noted every 60 s for 6 min using a VICTOR X3 device, PerkinElmer. Enzymatic activity was expressed as micromoles of NADPH utilized per minute and normalized to IU/g protein. The inter- and intra-assay CVs were 2.2 and 5.7%, respectively.

Glutathione S-transferase (GST) activity was assessed with Hebig and Jakoby's kinetic methods (38). In this method, 184 μl of reacting mixture along with GSH were added to 10 μl of homogenate. After initial reaction stabilization, 6 μl of 1-chloro-2,3-dinitrobenzene was dissolved in ethanol. The absorbance kinematics was observed at a wavelength of 355 nm every 60 s for 3 min with a VICTOR X3 device, PerkinElmer. Enzymatic activity was expressed as micromoles of thioether formed per minute and normalized to IU/g protein. The inter- and intra-assay CVs were 3.4 and 7.3%, respectively.

Total antioxidative capacity was determined with Erel's method (39). This method is based on oxidized colored 2,2'-azinobis-3-ethylbenzothiazoline-6-sulfonic acid radical cation (ABTS) decolorizing that is mediated by antioxidants present in the examined material. Absorbance was noted with the usage of the VICTOR X3, PerkinElmer with a 650 nm wavelength filter. The concentration was calculated on the basis of a model curve, while Trolox was used as a model. The values are presented as mmol/g protein. The inter- and intra-assay CVs were 1.1 and 3.8%, respectively.

#### Determination of Prooxidative Status Parameters

Oxidative stress in the gastrointestinal tract homogenates was determined with MDA, which was measured with Ohkawa et al.'s method (40). This was performed with a spectrofluorometer LS45 (PerkinElmer) at a wavelength 515 nm (absorbance) and 522 (emission). In contrast to spectrophotometric measurement, usage of a spectrofluorometer is more specific, as hemoglobin and bile pigments do not interfere with the measurement. This method has been modified by the addition of sodium sulfate and BHT, leading to an improvement in its specificity. Concentration of MDA was read on 1.1.3.3. tetraetoksypropan model curvature and presented as μmol/g protein. The inter- and intra-assay CVs were 2.2 and 8.4%, respectively.

Total oxidative stress (TOS) was assessed with the Erel method (41). The method is based on the oxidation of iron ions (II) into iron ions (III) in an acidic environment. Next, iron ions (III) with xylenol orange create a colored complex, achieving a purple/blue tone. Absorbance was measured with a 560 nm filter with the usage of a Victor X3, PerkinElmer. Concentration was calculated using an H_2_0_2_ model curve. Concentrations were expressed as μmol/g protein. The inter- and intra-assay CVs were 2.1 and 6.3%, respectively.

### Statistical Analysis

Statistical analysis was performed with Statistica ver.13 software. Quantitative variables were presented as a mean (*M*) with standard deviation (*SD*).

To compare the results obtained in the study groups with those obtained in control animals, a one-way ANOVA was performed. The homogeneity of variance was assessed with Leven's test. If homogeneity of variance was confirmed (*p* > 0.05), ANOVA with the least significant difference (LSD) *post-hoc* was used. If homogeneity of variance was not confirmed, a Kruskal–Wallis ANOVA with mean ranks *post-hoc* was then applied. The threshold for statistical significance was set at *p* < 0.05.

The study sample size was set with the use of G Power 3.1.94 software. With the assumption of effect size *f* = 0.50, α = 0.05, and statistical power (1 – β error probability) = 0.70, the sample size was established on 40 rats, showing an actual power of 0.709.

## Results

### Oxidative Stress Parameters in the Tongue Homogenates

**Figure 1:**
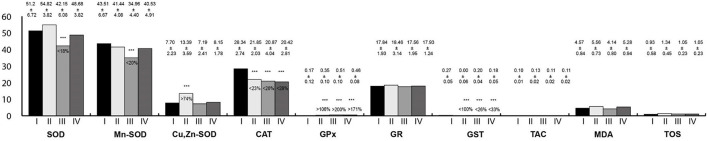
Oxidative stress parameters in the tongue homogenates. I, control group; II, group exposed to LF-EMFs; III, group exposed to RF-RMF; IV, group exposed simultaneously to LF-EMFs and RF-EMFs; SOD, superoxide dismutase (NU/mg protein); Mn-SOD, manganese-dependent superoxide dismutase (NU/mg protein); Cu,Zn-SOD, copper- and zinc-dependent superoxide dismutase (NU/mg protein); CAT, catalase (IU/g protein); GPx, glutathione peroxidase (IU/g protein); GR, glutathione reductase (IU/g protein); GST, glutathione S-transferase (IU/g protein); TAC, total antioxidant capacity (mmol/g protein); MDA, malondialdehyde **(**μmol/g protein); TOS, total oxidative status.

Oxidative stress parameters in the tongue homogenates are presented in Figure 1. CAT and GST activity were statistically significantly lower in the study groups than in the control. On the contrary, GPx activity was higher in the study groups. SOD and Mn-SOD activity were significantly lower in rats exposed to RF-EMFs in comparison with the control group. Then, Cu,Zn-SOD activity was statistically significantly higher in rats exposed to LF-EMFs than in the control group.

### Oxidative Stress Parameters in the Salivary Gland Homogenates

**Figure 2:**
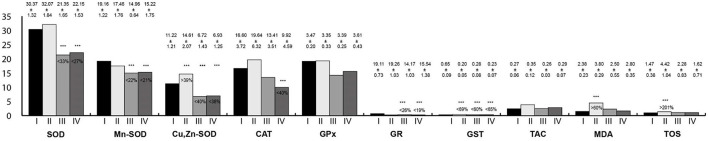
Oxidative stress parameters in the salivary gland homogenates. I, control group; II, group exposed to LF-EMFs; III, group exposed to RF-RMF; IV, group exposed simultaneously to LF-EMFs and RF-EMFs; SOD, superoxide dismutase (NU/mg protein); Mn-SOD, manganese-dependent superoxide dismutase (NU/mg protein); Cu,Zn-SOD, copper- and zinc-dependent superoxide dismutase (NU/mg protein); CAT, catalase (IU/g protein); GPx, glutathione peroxidase (IU/g protein); GR, glutathione reductase (IU/g protein); GST, glutathione S-transferase (IU/g protein); TAC, total antioxidant capacity (mmol/g protein); MDA, malondialdehyde **(**μmol/g protein); TOS, total oxidative status.

Oxidative stress parameters in the salivary gland homogenates are presented in Figure 2. Activity of SOD, Mn-SOD, Cu,Zn-SOD, GR, and GST in the salivary gland homogenates were statistically significantly lower in rats exposed to RF-EMFs and exposed simultaneously to LF-EMFs and RF-EMFs; whereas, the activity of Cu,Zn-SOD as well MDA and TOS concentrations in rats exposed to LF-EMFs were significantly higher in comparison with the control group.

### Oxidative Stress Parameters in the Esophagus Homogenates

**Figure 3:**
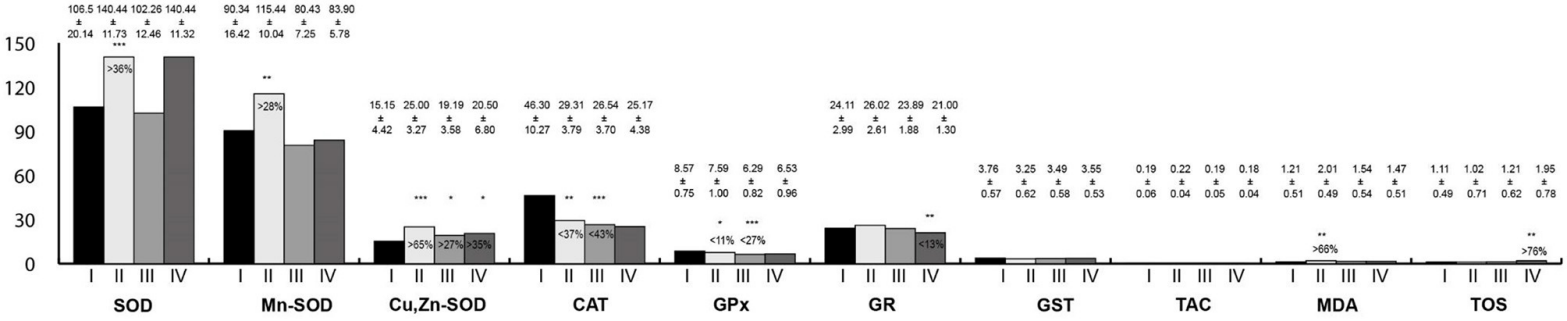
Oxidative stress parameters in the esophagus homogenates. I, control group; II, group exposed to LF-EMFs; III, group exposed to RF-RMF; IV, group exposed simultaneously to LF-EMFs and RF-EMFs; SOD, superoxide dismutase (NU/mg protein); Mn-SOD, manganese-dependent superoxide dismutase (NU/mg protein); Cu,Zn-SOD, copper- and zinc-dependent superoxide dismutase (NU/mg protein); CAT, catalase (IU/g protein); GPx, glutathione peroxidase (IU/g protein); GR, glutathione reductase (IU/g protein); GST, glutathione S-transferase (IU/g protein); TAC, total antioxidant capacity (mmol/g protein); MDA, malondialdehyde **(**μmol/g protein); TOS, total oxidative status. Differences statistically important at *p < 0.05; **p < 0.01; ***p < 0.001; *p*-value: particular study group vs. control group; *p*-value was added only if statistically significant. Percent values mean difference between study and control group; they were given if *p*-values were significant.

Oxidative stress parameters in the esophagus homogenates are presented in Figure 3. In rats exposed to LF-EMFs, the activities of SOD, Mn-SOD, Cu,Zn-SOD, and MDA concentration were statistically higher than in the control group; whereas CAT and GPx activities were lower. In the group exposed to RF-EMFs, Cu,Zn-SOD activity was higher than in the control group; however, CAT and GPx activities were lower. In animals exposed simultaneously to LF-EMFs and RF-EMFs, Cu,Zn-SOD activity and TOS concentration were higher than in control animals, whereas GR activity was lower.

### Oxidative Stress Parameters in the Stomach Homogenates

**Figure 4:**
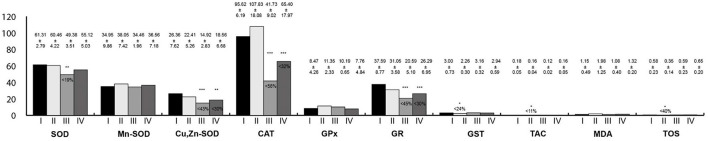
Oxidative stress parameters in the stomach homogenates. I, control group; II, group exposed to LF-EMFs; III, group exposed to RF-RMF; IV, group exposed simultaneously to LF-EMFs and RF-EMFs; SOD, superoxide dismutase (NU/mg protein); Mn-SOD, manganese-dependent superoxide dismutase (NU/mg protein); Cu,Zn-SOD, copper- and zinc-dependent superoxide dismutase (NU/mg protein); CAT, catalase (IU/g protein); GPx, glutathione peroxidase (IU/g protein); GR, glutathione reductase (IU/g protein); GST, glutathione S-transferase (IU/g protein); TAC, total antioxidant capacity (mmol/g protein); MDA, malondialdehyde **(**μmol/g protein); TOS, total oxidative status. Differences statistically important at *p < 0.05; **p < 0.01; ***p < 0.001; *p*-value: particular study group vs. control group; *p*-value was added only if statistically significant. Percent values mean difference between study and control group; they were given if *p*-values were significant.

Oxidative stress parameters in the stomach homogenates are presented in Figure 4. Activities of Cu,Zn-SOD, CAT, and GR were statistically significantly lower in the group exposed to RF-EMFs as well as in the group exposed simultaneously to LF-EMFs and RF-EMFs in comparison with the control. Furthermore, GST activity as well TAC and TOS concentrations were lower in rats exposed to LF-EMFs than in the controls.

### Oxidative Stress Parameters in the Small Intestine Homogenates

**Figure 5:**
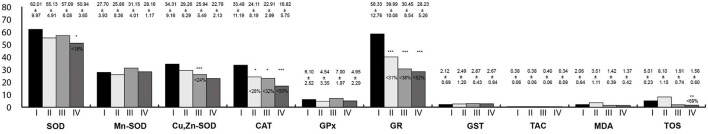
Oxidative stress parameters in the small intestine homogenates. I, control group; II, group exposed to LF-EMFs; III, group exposed to RF-RMF; IV, group exposed simultaneously to LF-EMFs and RF-EMFs; SOD, superoxide dismutase (NU/mg protein); Mn-SOD, manganese-dependent superoxide dismutase (NU/mg protein); Cu,Zn-SOD, copper- and zinc-dependent superoxide dismutase (NU/mg protein); CAT, catalase (IU/g protein); GPx, glutathione peroxidase (IU/g protein); GR, glutathione reductase (IU/g protein); GST, glutathione S-transferase (IU/g protein); TAC, total antioxidant capacity (mmol/g protein); MDA, malondialdehyde **(**μmol/g protein); TOS, total oxidative status. Differences statistically important at *p < 0.05; **p < 0.01; ***p < 0.001; *p*-value: particular study group vs. control group; *p*-value was added only if statistically significant. Percent values mean difference between study and control group; they were given if *p*-values were significant.

Oxidative stress parameters in the small intestine homogenates are presented in Figure 5. In all study groups, the activities of CAT and GR were lower than in the control group. Moreover, in animals exposed simultaneously to LF-EMFs and RF-EMFs, the activity of the SOD and TOS concentration were lower than in the controls. In rats exposed to RF-EMFs, Cu,Zn-SOD activity was lower than in the controls.

### Oxidative Stress Parameters in the Large Intestine Homogenates

**Figure 6:**
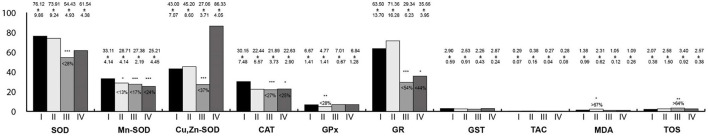
Oxidative stress parameters in the large intestine homogenates. I, control group; II, group exposed to LF-EMFs; III, group exposed to RF-RMF; IV, group exposed simultaneously to LF-EMFs and RF-EMFs; SOD, superoxide dismutase (NU/mg protein); Mn-SOD, manganese-dependent superoxide dismutase (NU/mg protein); Cu,Zn-SOD, copper- and zinc-dependent superoxide dismutase (NU/mg protein); CAT, catalase (IU/g protein); GPx, glutathione peroxidase (IU/g protein); GR, glutathione reductase (IU/g protein); GST, glutathione S-transferase (IU/g protein); TAC, total antioxidant capacity (mmol/g protein); MDA, malondialdehyde **(**μmol/g protein); TOS, total oxidative status. Differences statistically important at *p < 0.05; **p < 0.01; ***p < 0.001; *p*-value: particular study group vs. control group; *p*-value was added only if statistically significant. Percent values mean difference between study and control group; they were given if *p*-values were significant.

Oxidative stress parameters in the large intestine homogenates are presented in Figure 6. The activity of Mn-SOD was statistically lower in all study groups than in the control. In rats exposed to RF-EMFs, SOD, Cu,Zn-SOD, CAT, and GR activities were lower than in the control group, whereas TOS concentration was higher. Moreover, in rats exposed to LF-EMFs, the activity of GPx was lower whereas MDA concentration was higher in reference to the control animals. In rats exposed simultaneously to LF-EMFs and RF-EMFs, the activities of CAT and GR were lower than in the control group.

## Discussion

The major finding of our study is that a 4-week exposure to electromagnetic fields with features similar to those acting in the environment influences redox balance in the examined tissues. What is more, changes in the oxidative stress parameters are divergent and depend on the physical properties of the applied electromagnetic fields.

To our knowledge, this is the first study assessing the influence of common man-made electromagnetic fields on redox balance in the gastrointestinal tract. Available reports are focused mainly on the impact of LF-EMFs and RF-EMFs on nervous systems, which is a consequence of high brain sensitivity and susceptibility to oxidative injuries claimed to play a key role in several neuronal disorders (25). It is well-established that LF-EMFs and RF-EMFs increase the permeability of the blood–brain barrier. This may lead to excessive iron ions cumulating in the brain. Hydrogen peroxide reacts easily with iron ions, resulting in the synthesis of hydroxyl radical which oxidizes all molecules present in the cell (42). Nevertheless, mechanisms responsible for the activation of reactive oxygen species mediated by LF-EMFs and RF-EMFs remain unestablished. Similar to other body cells, it is triggered by the electron transport chain and nicotinamide adenine dinucleotide phosphate (NADPH) oxidases. In mitochondria, the electron transport chain is characterized by a physiological leakage of 2–5% of oxygen as a superoxide anion. On the other hand, NADPH oxidases constitute a group of membrane enzymes responsible for the secretion of superoxide radical anion during the transportation of electrons from NADPH to oxygen (43). However, further studies ought to discover the source of reactive oxygen species mediated by electromagnetic fields.

In the homogenates of the tongue, multidirectional changes of antioxidative markers were observed. An electromagnetic-field-mediated action decreased the activity of CAT, SOD, and Mn-SOD with a parallel increase in the concentration of GPx; it may be a consequence of enhanced production of hydrogen peroxide in the tissues of the tongue. Hydrogen peroxide is synthesized as a result of SOD-mediated superoxide anion radical dismutation, and then, it is transformed by GPx and CAT. A low level of hydrogen peroxide triggers its utilization by GPx through a reduced form of glutathione, whereas at high levels of hydrogen peroxide, this reaction is mediated by CAT (44). Diminished activity of CAT may indicate an enhanced production of hydrogen peroxide in tongue homogenates as a consequence of the electromagnetic field action. Despite the fact that the activities of CAT, SOD, and Mn-SOD were reduced in tongue tissues, an uncontrolled increase in the number of reactive oxygen species was not observed.

Salivary glands play a key role in homeostasis of the oral cavity. It is worth mentioning that periodontal inflammation is a primary reason for redox balance disturbances in the oral cavity (45). Bad dietary habits (46–48), chronic hyperglycaemia (49), and acute pancreatitis (50) are also well-recognized factors that significantly impede redox balance. Our study revealed that exposure to electromagnetic fields has a negative influence on redox milieu in the salivary glands, as was observed in rats exposed to LF-EMFs.

Barrett's esophagus and esophageal adenocarcinoma are mostly analyzed in the context of redox balance (51). In rats exposed to LF-EMFs, levels of lipid peroxidation, SOD, and its coenzymes were increased, whereas the activities of CAT and GPx were diminished. Enhanced activity of SOD and its coenzymes had to compensate for the production of superoxide radical anion. As a result, it led to a synthesis of the hydrogen peroxide, which had been utilized by CAT and GPx with a subsequent diminishment of their reserves. Moreover, lipid peroxidation might run in two mechanisms. First, in the presence of iron and copper ions, hydrogen peroxide initiates the production of hydroxyl radical which is claimed to be the strongest oxidant. Second, hydrogen peroxide may oxidize unsaturated fatty acids, leading to lipid membrane damage (44).

In stomach homogenates, statistically significant differences were observed only in the group of rats exposed to LF-EMFs. In the animals included in that group, levels of TOS, TAC, and GST were lower than in the control group. Except for GST, concentrations of other antioxidant enzymes did not change significantly. Probably, reactive oxidative species were removed by low molecular weight antioxidants with a secondary decrease of total antioxidant capacity. Indeed, concentration of the reduced form of glutathione is higher in the gastric mucosa in comparison with the remaining gastrointestinal tract sections (51). It may react with endo- and exogenous substances; those reactions are mediated by GST (52), and thus, its reserves might have been reduced.

In rats exposed simultaneously to LF-EMFs and RF-EMFs, the decreased level of total antioxidant status in the small intestine was a consequence of glutathione activity. Indeed, in that group of animals, the concentration of GR was statistically lower in comparison with the control group. The main aim of GR activity is to reset the reduced form of glutathione (52). In this study, we did not assess the levels of glutathione (GSH) and glutathione disulphide (GSSG); however, we measured indirect markers of GSH activity. In further studies, it is reasonable to take into consideration electromagnetic-field-mediated changes of interactions between GSH and GSSG, because it has already been proven that electromagnetic fields influence GSH level (53).

In the large intestine of rats exposed to LF-EMFs, levels of lipid peroxidation markers were higher than in the control group. However, in rats exposed to RF-EMFs, TOS was higher in comparison with control animals. It can be assumed that in the large intestine, the capacity to utilize reactive oxygen species was exceeded in the rats exposed to electromagnetic fields. Recent studies have reported that a high dosage of dietary iron (54), carbon irradiation (55), and organophosphate pesticide (56) may be included into external factors determining the redox balance.

This study proves that prooxidative process enhancement was the highest in the gastrointestinal tract of rats exposed to LF-EMFs. Despite the fact that magnetic field interference would be expected in a group exposed simultaneously to LF-EMFs and RF-EMFs, in homogenates of the esophagus a significantly higher concentration of TOS was observed in comparison with the control group. Moreover, MDA concentration in all examined tissues was comparable with the control group. Doubtless, in rats exposed simultaneously to LF-EMFs and RF-EMFs, reactive oxygen species activity was effectively compensated by antioxidative systems. Indeed, in that group as well as in rats exposed to RF-EMFs, numerous disorders in antioxidant activity were observed. Potentially, extension of exposure might lead to more significant changes in the prooxidative system due to the exhaustion of antioxidative reserves. It might also be reasonable to examine additional peroxidation markers. Without a doubt, our study illustrates the complex character of magnetic field action on biological systems.

This study has some limitations. First, we do not know the influence of magnetic-field-mediated redox balance changes on the function and cellular ultrastructure in digestive tract tissues. Second, we do not know if the observed reactions are a consequence of the direct action of magnetic fields on gastrointestinal tract tissues or are a result of whole-body exposure. Third, we examined the long-term effects of magnetic field action on digestive tract tissues. We do not know the short-term effects. Fourth, our study was carried out on an animal model; thus we are unable to indicate direct clinical implications. We do believe that our observations will start a new research direction, and further studies will be focused on the establishment of mechanisms of action and the potentially harmful effects of electromagnetic field action on the occurrence of gastrointestinal tract pathologies.

## Conclusions

Summing up, among those applied in the study, electromagnetic fields of a low-frequency caused the most significant disturbances of oxidative stress in the rat gastrointestinal tract. Chronic exposure to such electromagnetic fields should be listed as a potential risk factor for gastrointestinal tract pathologies.

## Data Availability Statement

The raw data supporting the conclusions of this article will be made available by the authors, without undue reservation.

## Ethics Statement

The animal study was reviewed and approved by Bioethical Committee for Animal Experimentation of the Medical University of Silesia in Katowice, Poland.

## Author Contributions

KS, AS, SK, EB, and ER contributed to conception and design of the study. KS organized the database. KS, KK, and GO performed the statistical analysis. KK wrote the first draft of the manuscript. KS, EB, ER, SK, GO, and AS wrote sections of the manuscript. All authors contributed to manuscript revision, read, and approved the submitted version.

## Funding

This study was supported by grant no. N N511351737 from the Ministry of Science and Higher Education.

## Conflict of Interest

The authors declare that the research was conducted in the absence of any commercial or financial relationships that could be construed as a potential conflict of interest.

## Publisher's Note

All claims expressed in this article are solely those of the authors and do not necessarily represent those of their affiliated organizations, or those of the publisher, the editors and the reviewers. Any product that may be evaluated in this article, or claim that may be made by its manufacturer, is not guaranteed or endorsed by the publisher.
